# Backward Dependencies and *in-Situ wh*-Questions as Test Cases on How to Approach Experimental Linguistics Research That Pursues Theoretical Linguistics Questions

**DOI:** 10.3389/fpsyg.2017.02237

**Published:** 2018-01-11

**Authors:** Leticia Pablos, Jenny Doetjes, Lisa L.-S. Cheng

**Affiliations:** ^1^Leiden University Centre for Linguistics, Leiden University, Leiden, Netherlands; ^2^Leiden Institute for Brain and Cognition, Leiden University, Leiden, Netherlands

**Keywords:** backward dependencies, *in-situ wh*-questions, coreference, negative polarity items, event-related potentials, self-paced reading, parsing, grammatical constraints

## Abstract

The empirical study of language is a young field in contemporary linguistics. This being the case, and following a natural development process, the field is currently at a stage where different research methods and experimental approaches are being put into question in terms of their validity. Without pretending to provide an answer with respect to the best way to conduct linguistics related experimental research, in this article we aim at examining the process that researchers follow in the design and implementation of experimental linguistics research with a goal to validate specific theoretical linguistic analyses. First, we discuss the general challenges that experimental work faces in finding a compromise between addressing theoretically relevant questions and being able to implement these questions in a specific controlled experimental paradigm. We discuss the Granularity Mismatch Problem (Poeppel and Embick, 2005) which addresses the challenges that research that is trying to bridge the representations and computations of language and their psycholinguistic/neurolinguistic evidence faces, and the basic assumptions that interdisciplinary research needs to consider due to the different conceptual granularity of the objects under study. To illustrate the practical implications of the points addressed, we compare two approaches to perform linguistic experimental research by reviewing a number of our own studies strongly grounded on theoretically informed questions. First, we show how linguistic phenomena similar at a conceptual level can be tested within the same language using measurement of event-related potentials (ERP) by discussing results from two ERP experiments on the processing of long-distance backward dependencies that involve coreference and negative polarity items respectively in Dutch. Second, we examine how the same linguistic phenomenon can be tested in different languages using reading time measures by discussing the outcome of four self-paced reading experiments on the processing of *in-situ wh*-questions in Mandarin Chinese and French. Finally, we review the implications that our findings have for the specific theoretical linguistics questions that we originally aimed to address. We conclude with an overview of the general insights that can be gained from the role of structural hierarchy and grammatical constraints in processing and the existing limitations on the generalization of results.

## Introduction

The study of language from an experimental point of view is a relatively young field in linguistics. In particular, work connected to the *parsing* or *on-line comprehension* of sentences—our area of interest in the present research—dates back to the late 60's and early 70's and has evolved from the work of various researchers who tried to put some of Chomsky's (1965) seminal ideas to test (e.g., Bever, 1970; Levelt, 1970; Kimball, 1973; Fodor et al., 1974; among others). Leaving the origins of the field aside (see Townsend and Bever, 2001; Phillips, 2013, for an overview), in this article we discuss the approach that researchers addressing topics based on strong theoretical linguistics background have taken to conduct experimental research that provides evidence for the validity of specific theoretical questions in linguistics or for the adequacy of general properties of language, such as structural hierarchy, or dependencies.

We first discuss the challenges this type of experimental approach faces in finding a balance between addressing theoretically relevant questions and being able to implement these questions in a controlled and realistic experimental paradigm. Secondly, we discuss the fact that certain theoretical questions can only be approached after building upon the evidence provided by a series of consecutive previous studies. Several researchers in the field have targeted a specific linguistic question starting from a seemingly simple paradigm in order to build upon the results and create more linguistically complex testing scenarios over thematically related follow-up experiments. Third, we illustrate through our own work two possible ways to carry out linguistic experimental research that bears heavily on linguistic theory. On the one hand, we examine linguistic phenomena that are similar at the conceptual level but different in their specific instantiations by investigating long-distance dependencies that involve either coreference of a cataphoric pronoun, or the backward interpretation of a negative polarity item in Dutch. These two linguistic phenomena have in common that the licensee always precedes its licensor and that the cue for how to identify a licensor rests upon the hierarchical structure. Specifically, we test how the expectation for the upcoming licensor might be impacted differently by linear and structural distance. For this, we discuss two experiments by Pablos et al. (2015, submitted) using event-related potentials (ERPs). On the other hand, we examine processing of a single linguistic phenomenon in unrelated languages. Specifically, we test the on-line processing of *wh*-*in-situ* questions in Mandarin Chinese and French. Current theoretical approaches all posit a dependency between the left periphery (e.g., in CP) and the *in-situ wh*-phrase, regardless of whether the dependency is established through covert movement of the *wh*-phrase to the left periphery or binding of the *wh-*phrase by a question-operator (for an overview, see Cheng, 2009; Bayer and Cheng, 2017). In processing terms, the parser does not encounter an overt cue to determine the interrogative or declarative nature of the upcoming structure until the *wh*-phrase position. At the *wh-*phrase position, the parser might need to backtrack to the left periphery to establish a dependency in order to interpret the *wh*-word. In relation to this second phenomenon, we discuss four self-paced reading experiments by Pablos et al. (submitted). Throughout the presentation of these two cases, we discuss the potential cost of simplifying a theoretically-based research question so that the empirical research can still lead to a meaningful contribution to linguistic theory. In particular, in the section Studies on the neural architecture of language we discuss how the research question can evolve from its starting point to its end point so that it becomes an empirically testable question.

### Challenges for theoretically informed experimental research in linguistics

In general, theoretical models are posited to represent the relationships, rules, constraints, etc., that relate different linguistic entities and structures. These theoretical models tend to rely mostly on evidence coming from speakers' judgment data and from corpus data. As it will be discussed in the section Studies on the neural architecture of language, there is an ongoing debate about whether the processing of language possesses mental representations that can be directly mapped to existing theoretical models (for further discussion see Phillips et al., 2011; Lewis and Phillips, 2015; Kush et al., 2017; Parker and Phillips, 2017; among others). Based on the assumption that this mapping exists, there is a growing amount of experimental work that evaluates if existing theoretical models can be corroborated and put to test.

One of the first challenges for this type of experimental approach is finding a compromise between addressing a theoretically relevant question and being able to implement the question at hand in a controlled experimental paradigm that leads to interpretable data and credible evidence. As this approach is driven by a theoretical linguistic question, the process starts by carefully thinking of an appropriate experimental setup that can target the question in the best possible way. The choice of methodology is also dependent on the theoretical question, which means that more than one method can be considered initially. There is a core difficulty about proceeding in this manner: the simplification of the linguistic paradigm linked to the research question. In this simplification process, attention has to be paid to two things: the first is to test with limited variables in the interest of interpretable results, and the second is the permanence of the core theoretical question to the extent that is still relevant to the discussion in the field.

Consider the licensing contexts of Negative Polarity Items^1^ (NPIs) as an example of a hypothetical testing scenario where the main research question is to find real-time or brain signatures of different NPI licensing environments. We know from existing theoretical linguistics research that NPIs can be licensed in different types of syntactic-semantic environments (e.g., conditionals, questions, comparatives, negative structures, see Giannakidou, 2011 for a full description). Thus, if there is some correspondence between the competence that speakers have of the different NPI licensing contexts and the speakers' use of this knowledge in real-time, a possible research question that we could put forth is whether these different syntactic-semantic environments yield different processing effects or whether these effects can be unified in that, if tested, they could all result into similar brain or psycholinguistic/algorithmic signatures. However, there is one constraint, namely, it is quite challenging to test all possible licensing contexts in one go. Further, if we test all possible contexts with one single experiment, we might get un-interpretable data from the fact that there are too many factors at play that are difficult to control experimentally. We therefore might break the question down into first testing only those contexts where there is an overt licensor (such as negation) that precedes the NPI. This reduces the number of factors and allows for a more uniform set of experimental stimuli, in the sense that we can at least identify the impact of an overt licensor in the processing of NPI (sentences) on-line. Once there is enough experimental evidence coming from testing environments with an overt licensor and some consensus has been reached on how NPI licensing works online (e.g., similar brain or psycholinguistic signatures are elicited), more contexts can be introduced in the experimental repertoire and in future experimental research examining the real-time signatures of NPI licensing. Nevertheless, this will only be possible when effects due to the NPI not being licensed, for example, have been robustly replicated intra-linguistically and possibly using different experimental methods. If we turn to the research on NPI processing of approximately the last 20 years, we can see that this is precisely how researchers working on this particular research question have approached this problem. Work by Shao and Neville (1998), Saddy et al. (2004), Drenhaus et al. (2005), Vasishth et al. (2008), Xiang et al. (2009), Yurchenko et al. (2013), and Parker and Phillips (2016), just to name a few, has examined the processing of NPIs by first looking at very basic paradigms where the licensor (i.e., negation) was either absent or in an inaccessible position. From all the existing research, to our knowledge, only Drenhaus et al. (2007), Steinhauer et al. (2010) and Xiang et al. (2016) examined other licensing environments that did not require an overt licensor (i.e., *wh*-questions in Drenhaus et al., 2007; non-veridical contexts in Steinhauer et al., 2010; and emotive predicates in Xiang et al. 2016).

Furthermore, the existing studies illustrate a lack of broad cross-linguistic research in that, except for a few studies that have examined the incremental interpretation of NPI licensing in languages such as Basque (Pablos and Saddy, 2009; Pablos et al., 2011), Mandarin Chinese (Tsai et al., 2013), Dutch (Yurchenko et al., 2013), Italian (Vespignani et al., 2009), Spanish (Pablos, 2009), and Turkish (Yanilmaz and Drury, 2013), most of the existing psycholinguistic generalizations have been made based on experimental evidence coming mainly from languages such as English and German. Further, the on-line methods used vary from the use of ERPs, to eye-tracking, self-paced reading and speeded acceptability judgments, and the questions they targeted varied in nature. In all of the studies, the *resulting effect* reflects an increase of mental processing effort or an interference effect in retrieving an element from memory, but the *observable* is different depending on the method, and cannot be univocally linked to a particular neurological/psychological process (see discussion of Poeppel and Embick's, 2005, *Granularity Mismatch Problem* in the section Studies on the neural architecture of language). Therefore, only a few generalizations can be made based on the existing experimental evidence and these generalizations come mainly from research that has examined illusory licensing effects in NPI licensing contexts (see Parker and Phillips, 2016 for an overview of these effects in the psycho/neurolinguistics literature).

### Studies on the neural architecture of language

One of the recurrent questions in the current psycholinguistic and neurolinguistic literature is whether researchers assume a correspondence between grammar (or our language competence system) and the parser (or our language performance system). Under the assumption of this correspondence, these two systems are able to feed each other and are part of the same cognitive system. Without such correspondence, the two systems are assumed to work separately and to abide by different rules or processes (see Lewis and Phillips, 2015 for further discussion). The research discussed here assumes that we have one cognitive system that is in charge of handling both competence and performance. What researchers working in the field of cognitive neuroscience of language have tried to address is the need to find a compromise between the theoretical assumptions that linguists take for granted and how these assumptions might be concretely realized in neurological terms (or signatures) and how they should be interpreted (see Marantz, 2005, 2013; Poeppel and Embick, 2005; Poeppel, 2012; Poeppel et al., 2012; Embick and Poeppel, 2015). Embick and Poeppel (2015, p. 358) describe one by one the challenges of how to test in an integrated way “theories of the (psycho)linguistic type that make claims about the computations and representations that constitute grammar and aspects of language use (referred to as “Computational-Representational” (CR) Theories)” in relation to “theories that study the structure and function of the brain coming from the Neurobiology of Language (NB) and that are more implementational in character.” Further, they discuss how CR-type of theories are currently more fine-grained than the current theories on how the linguistic representations and computations are realized in the brain (NB-theories). Under Poeppel and Embick's (2005, p. 104) and Embick and Poeppel's (2015, p. 361) view, what makes the unification of these two theories challenging is the *Granularity Mismatch Problem* (GMP), which refers to the fact that linguistic and neurolinguistic studies of language operate with objects of different “conceptual granularity.” Linguistic computation involves a number of fine-grained distinctions and explicit computational operations, whereas neuroscientific approaches involve broader conceptual distinctions. In their words, “this mismatch prevents the formulation of theoretically motivated, biologically grounded, and computationally explicit linking hypotheses that bridge neuroscience and linguistics” Poeppel and Embick (2005, p. 104) and it makes it “difficult to establish CR/NB linking hypotheses because in general the study of how the brain computes what it computes in language is at present too coarse to link up meaningfully with the distinctions made on the CR side” (Embick and Poeppel, 2015, p. 59). Adopting the view that the development of CR theory is an essential step toward understanding NB, Embick and Poeppel (2015, pp. 360–361) suggest three different ways in which CR and NB could interplay. The first is Correlational Neurolinguistics, where CR theories of language are used to investigate the NB foundations of language and in which knowledge of how the brain computes is gained by capitalizing on CR knowledge of language. This, for instance is the type of approach that works linking theoretical and psycholinguistic work have followed (see the work by Phillips and Lau, 2004; Lewis and Phillips, 2015, for example). The second way is Integrated Neurolinguistics, where Correlational Neurolinguistics plus the NB perspective provide crucial evidence that arbitrates among different CR theories. In Integrated Neurolinguistics, it is the brain data that enriches our understanding of language at the CR-level, for example. Third and last, Embick and Poeppel (2015) suggest that there is an Explanatory Neurolinguistics way where, besides Correlational and Integrated Neurolinguistics, something about NB structure or function explains why the CR theory of language involves particular computations and representations but not others.

Research over the past 10 years on the neural signatures of language has looked for experimental evidence that could show the process of how the building up of minimal units (which ranged from constituents, to minimal phrases to morphemes) occurs in the on-line computation of language, and that could show one of the basic intrinsic properties that characterizes the language faculty, namely, hierarchical structure. Within this field of work, we can distinguish three different groups of studies: (i) those that looked at whether there is hierarchy at the sentential level and whether this can be captured in terms of brain-oscillations or specific activations in syntax-semantics related brain areas (e.g., ERP studies by Luo and Poeppel, 2007; Arnal et al., 2015; Ding et al., 2015; Nelson et al., 2017; fMRI studies by Pallier et al., 2011; Brennan et al., 2012); (ii) those that examined whether a hierarchy can be found at the word level by using either fMRI or MEG methods (e.g., Fruchter and Marantz, 2015; Fruchter et al., 2015) and (iii) those that examined the compositionality of incremental meaning using MEG methodology (e.g., Bemis and Pylkkänen, 2011; Pylkkänen et al., 2011).

The evidence coming from the first set of studies suggests that we build sentences in small constituents as we parse them incrementally and that our brain makes clear distinctions between random word lists and sentences with different constituent length, either in a more constrained (or custom made) traditional experimental setting, or in a more natural one (e.g., Brennan et al., 2012). The evidence from the second set of studies suggests that we are aware of the constituency within words in that they show differences between morphemes that hierarchically depend on the root of the word vs. those that do not. Finally, the third set of studies provides support for the construction of semantic composition starting from minimal linguistic phrases such as *red boat* and comparing them with non-compositional contexts such as a word list, e.g., *cup, boat*.

Even though the above studies have looked at different linguistic phenomena, they all seem to point to the building up of minimal linguistic units in the brain, whether we are examining minimal linguistic units at a word, phrase or sentence level. Through the use of different methods and from evidence coming from either brain oscillations or specific brain area activations, these studies have shown that there is a way to capture the representation of constituent structure in the brain. Further, all these studies have started from very simple experimental paradigms where they examined the most minimal possible linguistic interaction and they built upon their own previous results to get to robust evidence that can lead to potential generalizations about the neurobiology of language.

### Current test cases: two ways to conduct strongly theoretically informed experimental studies

To illustrate some of the points made above, we discuss two ways in which we approach theoretical questions in experimental terms. The first way concerns the processing of two different linguistic phenomena, coreference and negative polarity item licensing, that are conceptually similar. Both coreference and negative polarity licensing can involve long-distance backward dependencies, where the licensee or dependent element occurs linearly before its licensor (although this configuration is not necessary for any of the two phenomena). Theoretical studies treat backward dependencies the same way as forward dependencies as structural hierarchy is the only important factor rather than linear precedence. The reasoning behind both ERP experiments is to examine if the strategies employed by the parser in the online interpretation of these two types of backward dependencies are similar, despite the different nature of the relation between the dependent element and its licensor. Even though the exact nature of the dependencies is different, both dependencies are restricted by syntactic structure. In other words, in both types of dependencies, there are positions in which the licensor can occur and positions from which it is impossible to enter into a licensing relation with the licensee. The question with respect to parsing is whether these structural restrictions are taken into account during an on-line parsing task, and whether the two types of dependencies are similar in this respect. These two types of dependencies were tested in the same language, Dutch, using the same methodology (ERPs).

The second way concerns the processing of the same linguistic phenomena, *wh*-*in-situ* questions, in languages with two different question formation strategies. French has both *wh*-fronting and *in-situ wh*-question strategies and Mandarin Chinese only has the *in-situ wh*-question strategy. The reasoning behind the four self-paced reading experiments we discuss is two-fold. First, as discussed above, we aim to examine the lack of an overt cue for a dependency with the left periphery (either through movement or through binding by a question-operator), and whether the encountering of the *in-situ wh*-phrase leads to backtracking in order to interpret the *in-situ wh*-phrase. Further, we examine whether the parser adopts different parsing strategies depending on whether the language only has one single *wh*-question formation strategy (e.g., only *in-situ* in Mandarin), or two strategies (as in French). If the strategies employed by the parser in the on-line interpretation of *wh*-*in-situ* questions in these two languages are alike, we can claim that there is a universal heuristics for interpreting *in-situ* questions in real-time. On the other hand, if the strategies differ between the two languages, we must conclude that they depend on the question formation strategies that are available to native speakers. From a theoretical point of view, it is expected that regardless of the question formation options that each language contains, *in-situ wh*-questions should be parsed similarly, namely, they need to establish dependency in the left periphery. This hypothesis considers the scenario where the grammar and the parser proceed hand-in-hand. The alternative would be an approach that shows an asymmetry between what is expected by theoretical linguistics research and what the real-time evidence shows, where the predictions for the performance side of language would be based on experience or usage-based information. If results come up differently for the two languages, it would mean that the existence of more than one question formation strategy in a language might impact the process of interpreting *in-situ wh*-questions in real-time differently. In order to address these questions, and assuming that the grammar and the parser might be unified, we tested whether *wh*-*in-situ* questions are processed inherently slower than their declarative counterparts when there is no prosody or context helping the online interpretation of *wh*-*in-situ* questions in these languages. This is the result that the theoretical approaches will predict.

## Test case 1: event-related potential experiments on backward dependencies in Dutch

### Cataphoric pronoun dependencies: search for antecedents only in grammatically licit positions

The ERP experiment in Pablos et al. (2015) examined the processing of a backward dependency involving cataphoric pronouns, i.e., pronouns that linearly precede their antecedent. The restriction of pronominal reference can be captured under the principles of the Binding Theory (Chomsky, 1981) that indicates the configurations in which nominal elements can or cannot establish a coreferential relation. There are three Binding Principles, each of which concerns a different type of nominal element. Principle C restricts the distribution of Referential Expressions, including proper names such as *Mary*. This Binding Principle prohibits a Referential Expression (e.g., proper name) from being bound (Chomsky, 1981). We tested if the Binding Principle C constrains the on-line comprehension of pronoun-antecedent dependencies; in particular, whether antecedents are only interpreted in relation to the preceding pronoun in grammatically licit contexts (i.e., where no grammatical constraint is violated), as in the interpretation of *Mary* in relation to the cataphoric possessive pronoun *her* in (1). This scenario can be contrasted with a scenario in which establishing the antecedent-pronoun relation violates the Binding Principle C, as in (2). In such a case, the antecedent *Mary* and the pronoun *she* cannot be interpreted as referring to the same person in (2).

(1) **Her**_j_ sister could not drive the car in Moscow while **Mary**_j_ was visiting.

(2) **She**_i/*j_ could not drive the car in Moscow while **Mary**_j_ was visiting.

In order to examine whether a grammatical constraint such as Binding Principle C is applied online in (2) and not in (1) at the proper name *Mary*, the well-attested *Gender Mismatch Effect* (GMME) paradigm was used (e.g., Sturt, 2003; van Gompel and Liversedge, 2003; Kazanina et al., 2007; Yoshida et al., 2014). In this paradigm, the gender mismatch effect at the antecedent position *Mary* with respect to *his* in (3) provides evidence that the parser has tried to interpret the pronoun at the antecedent position in this context. The GMME effect is observed in behavioral studies in that longer reading times in the mismatch condition in (3) than in the match condition in (1) are obtained. Conversely, when the antecedent position in (4) is compared to (2), no reading time difference is detected since *Mary* is barred as an antecedent due to Binding Principle C.

(3) **His**_k_ sister could not drive the car in Moscow while **Mary**_j_ was visiting.

(4) **He**_i_ could not drive the car in Moscow while **Mary**_j_ was visiting.

Previous studies have tested these specific pronoun-antecedent configurations in English and they measured reading times via different behavioral methods (i.e., self-paced reading and eye-tracking). The ERP study by Pablos et al. (2015) that we discuss here examined what the neural reflections of the GMME were^2^ and whether the GMME could be cross-linguistically attested.

#### Paradigm selection and materials' design

Following the self-paced reading study by Kazanina et al. (2007), Pablos et al. (2015) created four different experimental conditions in Dutch to test the sensitivity of the parser to Principle C. As in (1) and (3), two “no-constraint conditions” where the pronoun could be linked to the antecedent were introduced. This is shown in the sentences in (5) and (6), which contain a possessive pronoun that either matches (*haar* - female) or mismatches (*zijn* - male) the linearly first antecedent *Suzanne*^3^.

(5)   **Haar**_j_                  teamgenoten kondigden   aan            dat
       *her*                       *team mates* *announced* *PTC*          *that*
       **Suzanne Jansen**_j_ zeer             hoog
       *Suzanne*  *Jansen*  *very*            *highly*
       gewaardeerd         werd,          maar           **Edward**_i_    meldde
       *appreciated*          *was*,           *but*              *Edward*_*i*_    *reported*
       niet                        de              exacte         waardering.
       *not*                        *the*             *exact*          *rating*
       ‘Her teammates announced that Suzanne Jansen was
        highly appreciated, but Edward did not report the
        exact rating.’

(6)   **Zijn**_i_                     teamgenoten  kondigden   aan           dat
       *his*                        *team mates*  *announced*  *PTC*          *that*
       **Suzanne Jansen**_j_ zeer               hoog  
       *Suzanne Jansen*   *very*              *highly*  
       gewaardeerd         werd,            maar           **Edward**_i_    meldde
       *appreciated*          *was*,             *but*               *Edward*    *reported*
       niet                       de                 exacte         waardering.
       *not*                       *the*                *exact*          *rating*
        ‘His teammates announced that Suzanne Jansen was
        highly appreciated, but Edward did not report
        the exact rating.’

The other two experimental conditions were labeled as “Principle C conditions” and contained a cataphoric nominative pronoun in feminine [*zij* in (7)] or masculine [*hij* in (8)] form. Due to Principle C, these pronouns cannot corefer with the antecedent *Suzanne* in the embedded clause.

(7)   **Zij**_i_  kondigde     aan   dat  **Suzanne** **Jansen**_j_ zeer  hoog
        *she* *announced* *PTC* *that* *Suzanne* *Jansen*   *very highly*
        gewaardeerd werd,
        *appreciated*  *was*
        maar **Monika**_i_ meldde    niet de  exacte waardering.
       *but*    *Monika*   *reported* *not* *the* *exact*  *rating*
       ‘She announced that Suzanne Jansen was highly
       appreciated, but Monika did not report the exact rating.’

(8)     **Hij**_i_    kondigde        aan     dat   **Suzanne   Jansen**_j_   zeer   hoog
          he   announced   PTC   that   Suzanne   Jansen   very   highly
          gewaardeerd   werd,
         *appreciated   was*,
         maar   **Edward**_i_   meldde     niet     de     exacte   waardering.
         *but     Edward   reported   not     the    exact    rating*
        ‘He announced that Suzanne Jansen was highly
        appreciated, but Edward did not report the exact rating.’

#### Results and discussion

Pablos et al. (2015) found a significant ERP amplitude difference between the no-constraint conditions in (5) and (6) at the position of the name *Suzanne* in the antecedent *Suzanne Jansen*. This difference appeared as an anterior negativity over the 300–420 ms time window, where the no-constraint mismatch condition in (6) was more negative than the no-constraint match condition in (5) at the antecedent position. Furthermore, no difference was observed in the ERP waveforms between the Principle C constrained conditions in (7) and (8).

The results from this ERP experiment on Dutch backward pronoun dependencies show that the gender mismatch results in an anterior negativity and that, unlike in forward pronoun dependencies, there is no elicitation of a P600^4^. The anterior negativity is interpreted to be connected to failure of meeting the expectation to find an antecedent that matches in gender with the pronoun at the antecedent position. The main conclusion that one can draw from the results is that the parser is sensitive to gender mismatch effects only when they occur in grammatically licit positions. The fact that this effect is not present in the Principle C conditions means that the parser respects structural constraints when interpreting sentences in an incremental manner.

### Backward negative polarity item (NPI) dependencies: search for licensors only in grammatically licit positions

Similar to the cataphoric pronoun experiment discussed in the section Cataphoric pronoun dependencies, a second ERP study (Pablos et al. submitted) tested the processing of another backward dependency, a dependency involving negative polarity items. In this experiment, the Dutch negative polarity item *ook maar iets* “anything” occurs linearly before its licensor *niet* “not.” Consider first a situation where the licenser precedes the licensee as in (9a), and compare it with a context where the NPI appears linearly before the licensor, similar to the cataphoric pronoun dependency case, as in (9b) (where the NPI appears in a sentential subject). As discussed by Hoekstra (1991) and Hoeksema (2000), the subordinate clause *Dat het meisje ook maar iets geleerd heeft* “that the girl has learned anything” in (9b) is within the scope of the matrix negation *niet* “not,” meaning that structurally it is in a position where the NPI can be licensed by negation^5^. This is not the case with the negation *niet* “not” in the subordinate clause in (9c), where the NPI *ook maar iets* “anything” has scope over the negation. In this case the negation is in a position that is too low to act as a licensor of the NPI.

(9)     a. Het   is   **niet**   waarschijnlijk   dat     het       meisje   **ook maar iets**
             *it      is   not   probable          that   the       girl        anything*
             geleerd    heeft.
             *learned   has*
          b. [Dat   het   meisje   **ook maar iets**   geleerd   heeft]   is   **niet**
              *that   the   girl   anything               learned   has     is   not*
              waarschijnlijk.
              *probable*
             ‘That the girl has learned anything is not probable.‘
          c. *[Dat   het   meisje   **ook maar iets**   **niet**   geleerd   heeft]   is
                *that   the   girl      anything            not    learned   has     is*
                waarschijnlijk.
                *probable*
                Intended: ‘That the girl has not learned anything is
                probable.’

The central question of this experiment was again if the parser respects grammatical constraints which would be apparent if the parser is sensitive to the hierarchical position of the licensor. The condition of “backward” NPI such as (9b) is an excellent condition to test this as we do not expect any licensor within the sentence subject, i.e., the *dat* “that”-clause, as shown in (9c). Furthermore, if we assume an incremental interpretation of the sentence in (9b), the only overt cue that the parser encounters linearly to determine that there cannot be a licensor for the NPI within the subordinate clause is the complementizer *dat* “that” and this should be enough to determine that the licensor can only occur in the main clause. The idea was that if we increase the linear distance at positions in the sentence where the parser does not expect a licensor [i.e., any position after the NPI within the *dat* “that”-clause, indicated by [A] in (10)], it should be less costly to integrate the upcoming material incrementally than if we increase the linear distance at positions in the sentence where the licensor is highly expected [i.e., any position after the main clause verb “to be,” indicated by [B] in (10)].

(10)    [Dat   het   meisje   **ook maar iets [A]**   geleerd heeft]   is
          *that   the   girl       anything                   learned has    is*
          **[B]**   **niet**  waarschijnlijk.
                *   not   probable*
          ‘It is not probable that the girl has learned anything.’

We define the processing cost following the basic assumptions of the *Dependency Locality Theory* (DLT) proposed by Gibson (1998). Gibson proposed that two types of costs could contribute to structural complexity in real-time parsing: the storage cost and the integration cost, which draw on the same pool of working memory resources. Storage costs refers to the cost of keeping an element actively stored in memory while it cannot be interpreted and while other information in the sentence is being processed. The integration cost, on the other hand, refers to the cost of integrating a syntactic prediction at the time it can be satisfied. Further, these costs are both affected by locality, which is measured in relation to the number of new discourse referents being processed^6^. With respect to the processing cost that we refer to when the licensor in (10) is finally parsed, we specifically refer to the integration cost, which in this sentence is connected to the integration of the NPI with the licensor at the time the prediction for the appearance of the licensor is finally met. In previous ERP studies (e.g., Fiebach et al., 2002; Phillips et al., 2005), this integration cost has been shown to elicit a P600 at the position where the syntactic prediction is met. Further, as noted in footnote 4, its amplitude has been shown to reflect the degree of difficulty of the syntactic integration at hand; therefore, one would expect that a higher integration cost will be shown in terms of differences in the amplitude of the elicited ERP component.

#### Paradigm selection and materials' design

In order to test the described contrast and implement the effects of increasing the linear distance between the NPI and negation (i.e., the licensor), Pablos et al. (submitted) introduced conditions that added one to two modifiers at either A or B positions in (10). These conditions were compared at the licensor position (i.e., negation) with a control such as (9b), where no additional material was introduced. As mentioned in the section that discusses the challenges for theoretically informed experimental research, the experimental paradigm must be carefully controlled to avoid introducing differences that can affect the results: the modifiers that were included always consisted of three words each and had no possible interference in the interpretation of the NPI besides delaying the appearance of negation^7^. In (11a) and (11b), we reproduce examples of the experimental materials with the modifiers that were included at the A position. Again, it was expected that this contrast would not result in a high integration processing cost (in the terms we defined above) at the licensor position (i.e., negation), as the modifiers 1 and 2 occur at a structural position where negation cannot appear.

(11)    a. Dat   het   meisje   **ook maar iets**   [over   dit   vak]_mod1_
              *that   the  girl      anything             about this subject*
              geleerd   heeft   is   **niet**   waarschijnlijk.
              *learned   has   is   not   probable*
              ‘It is not probable that the girl has learned anything
               about this subject.’
          b. Dat   het   meisje   **ook maar iets**   [over   dit   vak]_mod1_   [op
              *that   the   girl     anything             about this subject       at*
              de   universiteit]_mod2_   geleerd         heeft  is  **niet**
              *the university            learned         has   is   not*
              waarschijnlijk.
              *probable*
            ‘It is not probable that the girl has learned anything
            about this subject in the university.’

On the other hand, in (12a) and (12b) modifiers were added to the main clause B position, which occurs adjacent to the main verb “to be.” It was expected that this contrast would result in a higher integration cost at negation due to the modifiers occurring at a structural position where negation can appear.

(12)   a. Dat  het  meisje  **ook maar iets**  geleerd  heeft  is
              *that* *the* *girl*     *anything*            *learned* *has*    *is*
              [volgens         haar docent]_mod1_
              *according* *to* *her*   *lecturer*
              **niet** waarschijnlijk.
              *not* *probable.*
              ‘According to her lecturer, it is not probable that the girl
              has learned anything.’
          b. Dat het meisje **ook maar iets** geleerd heeft is
              *that* *the* *girl*   *anything*          *learned* *has*  *is*
              [volgens      haar docent]_mod1_
              *according* *to* *her*   *lecturer*
              [vanwege haar afwezigheid]_mod2_ **niet** waarschijnlijk.
              *due to*     *her*   *absence*              *not*      *probable.*
              ‘According to her lecturer, it is not probable that the girl
               has learned anything due to her absence.’

Due to the fact that the NPI appears within a sentential subject clause, it is highly probable that the licensor is a negation (and not other NPI licensing environments such as conditionals, questions, etc.). Relevantly, in comparison with previous studies, the additional modifiers do not turn the test sentence into an ungrammatical continuation but rather add just extra information, avoiding effects due to grammaticality that can confound the interpretation of the results.

There are two types of potential effects that should be differentiated in the above manipulations. One is an integration cost effect from the fact that the dependency started at the NPI has decayed and retrieval of the NPI from memory when the licensor is found would be costly, and the other is a facilitation effect from the fact that negation is highly expected (and wanted) at the time the licensor is encountered. The third effect is an effect connected to the actual incremental integration of the added modifiers and the fact that their integration also delays the appearance of the licensor (negation). Again, if the predictions we set in the section Backward Negative Polarity Item (NPI) dependencies were met, we do not expect any effect with added modifiers in the A position [as in (11a,b)], while effects are expected in the B position [as in (12a,b)]. Moreover, we expect to find an ERP component that is associated with syntactic integration costs and a difference in the amplitude of the ERP component to occur relative to the difficulty of integrating the syntactic prediction.

#### Results and discussion

Results confirm the expected contrast between the conditions in (11a) and (11b), and those in (12a) and (12b) at the negation position, when compared with their baseline condition in (9b).

The statistical analysis of the data confirmed the presence of a significant central anterior negativity in the 200–600 ms time window at the position of negation when the control sentence in (9b) was compared to conditions (12a) and (12b) at negation. When (9b) was compared to (11a) and (11b) conditions, only a lower, non-significant difference emerged. As expected, the amplitude of the negativity showed a correlation with the position and number of modifiers in the sentence with respect to the position of negation. When modifiers are introduced at the main clause following the verb *is* (i.e., position B), the amplitude of the central anterior negativity was bigger than when modifiers are introduced within the embedded clause after the NPI (i.e., position A). This shows that the parser is sensitive to structural positions in the sentence and that it considers the grammatical constraints for encoding the search for a location where a potential licensor for the NPI can occur. Furthermore, the results show that there is a different integration cost depending on the number of modifiers that are introduced at the potential licensor position.

While observable differences support the interpretation of the research question, the exact nature of the underlying process causing the ERP difference is questionable. Within the ERP literature in sentence comprehension, sustained negativities have been found for conditions that demanded a high memory load (e.g., Kluender and Kutas, 1993; King and Kutas, 1995; Friederici et al., 1996; Müller et al., 1997; Münte et al., 1998; Fiebach et al., 2002). In particular, they were found in studies that examined processing of dependencies of different lengths, where they manipulated linear distance from the start of the dependency to the closure point. These studies compared contexts of short vs. long-distance *wh*-questions (see Fiebach et al., 2002; Phillips et al., 2005) and object vs. subject relative clause contexts (King and Kutas, 1995). Furthermore, these studies carried out two types of analysis of the data. In the classic single-word ERP analysis they examined the ERPs at the beginning (i.e., *wh*-word or relativizer) and at the end of the dependencies (verb), whereas in the multiword ERP analysis of the data, they examined the ERPs elicited at each of the words of the dependency, from the beginning (e.g., *wh*-word) to the closure of the dependency (e.g. the verb)^8^.

In the data from Pablos et al. (submitted), we take the beginning of the dependency to be marked by the NPI (i.e., the licensee) and the end marked by negation (i.e., the licensor). The position of negation is therefore the position where the dependency can be completed or finally integrated. It might be reasonable to think that the observed central anterior negativity marks the overall integration of the licensor for the NPI in sentences when the licensor-licensee distance is longer relative to the control. The size of the ERP amplitude is taken to reflect the level of disruption that additional material can cause in the search for a licensor. The fact that the effect correlates with the position of the intervening material (i.e., its size is relative to the position where the licensor is most likely to occur) suggests that structural conditions play a role in this process. As discussed in the section on NPI dependencies, previous studies that examined short vs. long-distance *wh*-questions (see Fiebach et al., 2002; Phillips et al., 2005) have shown the elicitation of a P600 at the verb where the dependency is completed and have interpreted it as an integration cost related to the integration of the syntactic prediction. The fact that the type of dependency we examined is of a slight different nature (i.e., on the syntax-semantics interface) might have contributed to having a different type of ERP component elicited. Again, it should be emphasized that the study by Pablos et al. (submitted) does not examine cases of licensing failure as previous researchers have done in the experimental NPI literature. Instead, it looks at grammatical instances of NPI licensing where (a) the NPI occurs linearly preceding its licensor; and (b) what is manipulated is the delay of the occurrence of the licensor at different grammatical positions. This reasoning is a bit different in spirit from previous NPI research, but it allows us to draw a parallel between the two different kinds of backward dependencies presented in the section Test Case 1 in order to answer the question of whether the parser proceeds similarly in the strategies that it adopts when proceeding in the incremental interpretation of phenomena that occur long-distance.

### General discussion of experiments on test case 1

Summarizing the main results of the ERP experiments discussed within our first test case, we first showed that gender mismatch effects in sentences containing cataphora result in anterior negativities in the 300–420 ms time-window when the gender of the antecedent mismatches that of the pronoun in no-constraint conditions. We then observed that (a) the delay in the appearance of the licensor in a structure with fronted NPIs results in a central anterior negativity in the 200–600 ms time-window at the position of negation and (b) the difference in ERP amplitude size for the anterior negativity reflects an increased integration cost correlated with the structural position where a licensor is allowed to appear.

The common finding of these ERP experiments is that the parser respects the grammatical restrictions posited in the two configurations. In the case of coreference, the parser did not try to link the pronoun with potential antecedents in positions where the grammar (i.e., Binding Principle C) prohibits coreference, due to c-command, a hierarchical relation. In the case of NPI backward licensing, only modifiers added immediately before the grammatically licit licensor affect the processing of this licensor, again because the licensor position that matters is the one in which a potential licensor can have scope over the NPI, which is a necessary condition for licensing it. Even though we are not able to directly compare the elicited ERP components (since they are generated for different stimuli and their latencies and topographies do not overlap completely), these results point to the application of grammatical constraints in the on-line interpretation of the stimuli. This idea is on a par with Parker and Phillips (2016), where dependencies that consist of subject-verb agreement or reflexive-antecedents are said to deploy the same memory access mechanisms despite differing in cue weightings.

Furthermore, if we abstract away from the elicited specific ERP components, we can claim that these results yield evidence for the existence of basic hierarchical relations in parsing. These hierarchical relations are an intrinsic property of our language capacity, therefore, the results support a one-system architecture (Lewis and Phillips, 2015), where the grammar and the parser are part of the same cognitive system (as discussed in the section that has examined the neural architecture of language). Being part of the same cognitive system does not necessarily entail that the heuristics need to come in the same form in both grammar and parser, but it seems logical to assume that some of the basic properties, such as hierarchical relations, are indeed universal and shared by both. As discussed by Phillips et al. (2011) and Kush et al. (2017), one relevant property present in both the cataphora and the backward NPI licensing cases discussed within our first test case is the directionality of the dependency, where the left-hand element provides reliable information in the prospective search for an antecedent in cataphoric dependencies and for a licensor in NPI licensing dependencies.

## Test case 2: experiments on *wh-in-situ* questions in mandarin Chinese and French

As a second illustration of the points raised in the Introduction, in this section, we review a set of experiments where the same linguistic phenomenon is examined cross-linguistically to investigate the generalizability of parsing processes. The difference lays in the *wh*-question formation strategies available in the two tested languages.

French is a language that employs two different strategies for question formation. Even though *wh*-*in-situ* is an option (13b), it also allows various types of structures which involve *wh*-fronting as in (13a)^9^:

(13)   a. **Qui**   tu     as     vu ?
               *who* *you* *have* *seen*
             ‘Who have you seen?’
          b. Tu    as     vu    **qui** ?
               *you* *have* *seen* *who*
             ‘Who have you seen?’
          c. Marie a    vu     Jean.
              *Marie* *has* *seen* *Jean*
             ‘Marie has seen Jean.’

Whereas French has two different question formation strategies, Mandarin Chinese only has one, which we call the *in-situ wh*-question formation strategy. As shown in (14a), in this strategy the question word *shéi* “who” remains in its canonical position.

(14)   a. Ni   zuótiān     yùjiàn le       **shéi?**
             *you* *yesterday* *meet*  perf   *who*
             ‘Who did you meet yesterday?’
          b. Lĭsì zuótiān      yùjiàn le      Zhāngsān.
              *Lisi* *yesterday* *meet*  perf *Zhangsan*
             ‘Lisi met Zhangsan yesterday.’

As we can see in (13) and (14), in the case of *wh-in-situ* questions, the clause type of the sentence (question or declarative) is only apparent at the point the *wh*-word is encountered [as evidenced by the comparison between (13b) and (13c) and between (14a) and (14b)]. Crucially, no distinction can be made on the surface between these two sentences by readers as they process the sentence, unless there is prosodic or contextual information available. Therefore, sentences like those in (13b) and (14a) posit an interesting question with regard to parsing covert dependencies in that, if the sentence is read and it lacks any other kind of overt cue aiding its interpretation, there are different parsing heuristics that the parser might adopt.

The syntactic literature has claimed that although *in-situ wh*-questions have no overt movement, they are formed via a covert dependency, where the *wh*-word can either relate to the left periphery (where the clause type of the sentence is flagged) via operator-variable binding, or via covert movement at Logical Form (LF; for further discussion see Huang, 1982; Cheng, 1991, 2009; Aoun and Li, 1993; Tsai, 1994; Bayer and Cheng, 2017). The theoretical proposals differ in the means by which the covert-dependency is formed, but they share the core assumption that there is a higher position in the structure (i.e., SpecCP) where the clause type is marked. This in turn raises an interesting question with regard to their *representation* in the language processing system. Overt dependencies have been shown to trigger active search mechanisms as soon as a fronted *wh-*word is encountered (e.g., Crain and Fodor, 1985; Stowe, 1986), but the mechanism that the parser follows in interpreting *in-situ wh*-questions is not clear since there is no trigger (or cue) for a search for a *wh*-word/phrase. Therefore, the research questions that the current test case addresses are: (a) which are the processes involved in reading *in-situ wh-*questions where no overt trigger is present for the incremental buildup of the relevant dependency? and (b) which are the observable effects of establishing the dependency in the left periphery for the *wh*-phrase?

As a first attempt we can entertain two possible approaches for the processing of *in-situ wh*-phrases: (i) the parser always posits a covert dependency from the beginning of the sentence, and therefore postulates a silent structural position at the start of the parse, or (ii) the parser only realizes it needs to establish a covert dependency when it encounters the *in-situ wh*-word/phrase. If the parser adopts the first approach, there should not be any processing cost effect observable when comparing declarative and *wh-in-situ* questions, since both are equally considered from the beginning of the parse. With the latter strategy, at the *in-situ wh*-word position, the parser will realize that a covert *wh-*dependency needs to be established, whereas this would not be necessary in declarative constructions. This effect should be similar in both Mandarin and French.

Moving one step further, it might also be possible that the integration and processing cost (see Gibson, 1998) for the covert operator position in the left periphery of a sentence differs depending on whether the language only has an *in-situ* question formation strategy (like Mandarin), or whether it is optionally *in-situ* (like French). In a language like French, once the fronted *wh*-question possibility has been discarded, the *in-situ* question continuation possibility may be less entertained. In Mandarin, where the *in-situ* strategy is the only one, the parser may anticipate the possibility of having a covert question operator, and thus encounter fewer difficulties in integrating the *in-situ wh*-expression. Thus, a further research question is: to what extent is the parser able to anticipate the upcoming structure when there is no information available to determine the *likelihood* of encountering an *in-situ* question?

The study of the processing of covert dependencies in *in-situ wh*-questions in Mandarin Chinese has already been approached in the work of Xiang et al. (2013, 2015). Xiang et al. (2013, 2015) have examined the processing of *in-situ* questions with complex *wh*-phrases with two different dependency lengths (with one embedding vs. mono clausal) and declaratives that contained definite noun phrases using different methodologies (i.e., Speed Accuracy Trade-Off (SAT), self-paced reading and eye-tracking). Xiang et al. (2013, 2015) found that *in-situ wh*-questions were processed slower, especially when *in-situ wh*-questions with one embedding were compared with mono-clausal questions. Nevertheless, there are still some questions that remain concerning the generalizations that we can make regarding the processing of *in-situ wh*-questions. This is so because in the psycholinguistics literature both complex *wh*-phrases and definite noun phrases have been claimed to involve higher processing cost, that is, connected to the increase of the complexity of the parse, as we have discussed in the section on NPI dependencies (see also footnote 6). In complex *wh*-phrases, for example, the processing cost is said to be attributed to the discourse-linking nature of these *wh*-phrases (see De Vincenzi, 1996; Kaan et al., 2000; Donkers et al., 2013), whereas in the case of definite noun phrases, the processing cost is due to the fact that they refer to discourse entities that are less accessible and to their position in the Accessibility Hierarchy (see Ariel, 1990; Gundel et al., 1993; Warren and Gibson, 2002). Furthermore, since there is theoretical research showing that *wh-*words are closer to indefinites (see Huang, 1982; Cheng, 1991, among others), the self-paced reading experiments we report here addressed these issues connected to syntactic complexity by including an additional comparison between declarative sentences with definite and indefinite noun phrases with questions, in contexts where the *wh*-phrase was simplex (*qui* “who” and *shéi* “who”) or contexts where the *wh*-phrase was complex (such as *quel ami* “which friend” in French and *nǎgè péngyǒu* “which friend” in Mandarin Chinese).

In testing the phenomenon of *in-situ wh*-questions, Pablos et al. (submitted) wanted to compare how the incremental reading of *in-situ wh*-questions proceeds in comparison to the reading of their declarative counterparts that contain the exact same content up to the *wh*-word/noun phrase position. Their aim was two-fold: first, they wanted to investigate if the *wh*-word/phrase is expected, and if its integration is expected to be without any additional cost in comparison to its declarative counterpart; and second, they wanted to investigate whether the available *wh*-question formation strategies in each language have an impact on the initial hypotheses that are being considered by the parser before the *wh*-word/phrase position is encountered. The next section discusses the results of the four reading time experiments in Pablos et al. (submitted) on the processing of *wh*-*in-situ* questions in French and Mandarin Chinese.

### Processing simplex *wh*-*in-situ* questions in French

The first of the four self-paced reading experiments in Pablos et al. (submitted) examined the contrast shown in (15) in order to test whether reading time differences can be found between questions and declaratives. To limit spurious effects, care was taken in the design of the materials: (i) the *wh*-word *qui* “who” in (15a) and the indefinite noun phrase *quelqu'un* “someone” in (15b) remain constant throughout the whole experiment; (ii) in the definite noun phrase condition, mono- and disyllabic proper names were used^10^ to provide a match both with the length of the *wh*-word *qui* and the indefinite noun phrase *quelqu'un*, as illustrated in (15c); (iii) all other elements among conditions were kept minimally different.

(15)   a. *In-situ question with a simplex wh-phrase*
             Le braqueur de banque a blessé **qui**      dans sa
             *the robber*   *of bank*    *has hurt* *whom on*   *his*
             fuite ?
             *escape*
             ‘Who has the bank robber hurt on his escape?’
          b. *Declarative with* *indefinite* *object noun phrase*
              Le braqueur de banque a     blessé **quelqu’un** dans sa
              *the robber*   *of bank*    *has hurt*    *someone*    *on*   *his*
              fuite.
              *escape*
             ‘The robber of the bank has hurt someone on his
              escape.’
          c. *Declarative with* *Proper* *Name object*
             Le braqueur de banque a    blessé **Marie/Jean** dans sa
             *the robber*   *of bank*    *has hurt*   *Marie/Jean*   *on*   *his*
             fuite.
             *escape*
             ‘The robber of the bank has hurt Marie/Jean on his
             escape.’

Considering the predictions of the two possible parsing approaches described above, if only a declarative interpretation was assumed from the beginning of the sentence, the parser would need to reanalyze its initial assumption, which in turn will result in reading time differences between the declarative sentences in (15b) and (15c) and the question in (15a) at the *wh*-word/noun phrase position. Conversely, if the parser considers in parallel both possible interpretations, no reading time differences are expected between the question and the declarative conditions.

Comparison of the residual reading times of the sentences in (15) shows that there is a difference in processing times between declaratives and *in-situ* questions with a simple *wh*-phrase starting from the *wh*-word/noun phrase position. The timing of this difference depends on the type of declarative. When it contains an indefinite such as *quelqu'un* “someone” in (15b), the difference between questions and declaratives occurs as soon as the *wh*-word is encountered, whereas when it contains a proper name such as *Marie* in (18c), this difference only occurs once the proper name has been interpreted at the immediately following region [i.e., the preposition *dans* “in” within the examples in (15)].

### Processing complex *wh*-*in-situ* questions in French

The second experiment examined the processing of questions and declaratives containing complex *wh*-phrases/noun phrases. The stimuli followed the form of the simplex *wh*-question experiment, where changes between the two experiments were only implemented at the *wh*-phrase/noun phrase position. An example of a set of materials is given in (16), with a complex *wh*-phrase *quelle caissière* “which cashier” in the *wh* condition in (16a), declaratives with an indefinite noun phrase *une caissière* “a cashier” in (16b) and declaratives with a definite noun phrase *la caissière* “the cashier” in (16c).

(16)   a. *In-situ question with a complex wh-phrase*
             Le braqueur de banque a   blessé **quelle caissière**
             *the robber*   *of bank*    *has hurt*   *which cashier*
             dans sa  fuite ?
             *in     his* *escape?*
             ‘Which cashier has the bank robber hurt on his escape?’
          b. *Declarative with* *indefinite* *object noun phrase*
              Le braqueur de banque a    blessé **une caissière** dans
              *the* *robber*   *of bank*    *has hurt*    *a*    *cashier*     *in*
              sa  fuite.
              *his* *escape.*
             ‘The bank robber hurt a cashier on his escape.’
          c. *Declarative with definite* *object noun* *phrase*
              Le braqueur de banque a    blessé **la**    **caissière** dans
              *the* *robber*   *of bank*    *has hurt   the   cashier    in*
              sa   fuite.
              *his* *escape.*
              ‘The bank robber has hurt the cashier on his escape.’

The same predictions about the two possible parsing approaches described in the case of simplex *wh-*phrases are applicable to the comparison between complex *wh-*phrases and declaratives. Our results show that *in-situ* questions with a complex *wh-*phrase such as *quelle caissière* “which cashier” in (16a) are again significantly slower to read than declaratives that contain an indefinite noun phrase such as *une caissière* “a cashier” in (16b). Interestingly, this effect is not apparent until the whole *wh*-phrase has been processed, since the effect appears at the word immediately after the *wh*-phrase (i.e., the preposition *dans* “in”). Note that *quel(le)* is the determiner of the interrogative *wh*-phrase, and the participants clearly waited until the end of the *wh*-phrase. Furthermore, given that noun phrases in French can have post-nominal modification (*quelle caissière* *débordée* “which overworked cashier” or *quelle caissière* *de supermarché* “which grocery store cashier”), the effect might be due to readers not considering the *wh-*phrases to be completed until they reached the region immediately following the noun.

#### General discussion of experiments on processing French *in-situ wh-*questions

Results from both the simplex and complex *in-situ wh*-questions in French showed that questions containing both type of *wh*-phrases are generally processed slower than their declarative counterparts, in particular those declaratives that contain indefinite noun phrases such as *quelqu'un* “someone” in (15b) and *une caissière* “a cashier” in (16b). We discuss the implications of these findings in connection to those of the self-paced readings on Mandarin Chinese in the general discussion of experiments on test case 2.

### Processing simplex *wh*-*in-situ* questions in mandarin Chinese

The same paradigm as in the French experiment described in the section Processing Simplex *wh*-*in-situ* Questions in French was used in Mandarin Chinese by Pablos et al. (submitted), contrasting *wh*-*in-situ* questions with declarative sentences. In the object position, the *wh*-word *shéi* “who” was used in the *wh*-questions and *rén* “someone” (indefinite) or a proper name (definite) in declaratives.

Again, three conditions were designed to test whether reading time differences can be found between questions and declaratives in Mandarin Chinese. As in French, care was taken to minimize the differences between conditions to avoid unintentional bias of the results due to uncontrolled effects: (i) The *wh*-word *shéi* “who” in (17a) and the indefinite noun phrase *rén* “someone” in (17b) are monosyllabic and they were not changed throughout the whole experiment, whereas the proper names in (17c) were varied in having different bisyllabic proper names all throughout^11^; (ii) in order to make sure that the indefinite *rén* “someone” had only the indefinite interpretation available, intensional verbs were used and the perfective marker –*le* was omitted (see Cheng and Sybesma, 1999 for further discussion); (iii) the use of intensional predicates allowed for two extra regions after the *wh*-word/phrase position, which occurs usually sentence finally in Mandarin Chinese, to avoid confounds of sentence wrap-up effects at the *wh*-word position.

(17) a. *In-situ question with a simplex phrase*
        那个 男生       想要      求  谁    解决 问题?
        Nàgè nánshēng xiǎngyào qiú   **shéi** jiějué wèntí?
        *the*^12^ *boy         want       beg who solve problem*
        ‘Who did the boy want to beg to solve the problem?’
        b. *Declarative with indefinite object noun phrase*
        那个  男生       想要      求  人
        Nàgè  nánshēng xiǎngyào qiú   **rén**
        *the     boy          want       beg person*
        解决  问题.
        jiějué  wèntí.
        *solve problem*
        ‘The boy wants to beg someone to solve the problem.’
        c. *Declarative with Proper Name object*
        那个 男生       想要      求  小张
        Nàgè nánshēng xiǎngyào qiú   **Xiǎozhāng**
        *the    boy         want       beg Xiaozhang*
        解决 问题.
        jiějué wèntí.
        *solve problem*
        ‘The boy wants to beg Xiaozhang to solve the problem.’

The results of *in-situ* questions with a simplex *wh*-phrase in Mandarin Chinese show that *in-situ* questions with a simplex *wh*-phrase [*shéi* “who” in (17a)], were read significantly slower than their indefinite declarative counterparts [*rén* “person/someone” in (17b)] immediately after the *wh*-word, at the verb *jiejué* “to solve.” However, at the *wh*-word position *shéi* “who” in (17a), *in-situ* questions are significantly faster than their Proper Name counterparts in (17c). This slowdown effect at the proper name is attributed to two possible reasons:

(1) proper names in the experiment materials having two morphemes/syllables while the question word *shéi* “who” only has one^13^ and (2) the processing of proper names in Mandarin Chinese has been shown to be more costly than the processing of common nouns (see Yen, 2007).

### Processing complex *wh*-*in-situ* questions in mandarin Chinese

The fourth and final experiment in Mandarin Chinese from Pablos et al. (submitted) used the same paradigm as the French experiment that tested the processing of complex *wh-in-situ* questions in French, by contrasting *wh*-*in-situ* questions with declarative sentences. The stimuli followed the form as the simplex *wh*-question experiment described in the previous section. It only differed in content at the position of the *wh*-phrase/noun phrase: a complex *wh-in-situ* constituent [e.g., *nǎgè tóngxué* “which classmate” in (18a)] was contrasted with complex noun phrases of two types [e.g., the indefinite *yígè tóngxué* “a classmate” in (18b) and the definite *nàgè tóngxué* “the classmate” in (18c)].

(18) a. *In-situ question with a complex phrase*
        那个  男生      想要      求  那个 同学
        Nàgè nánshēng xiǎngyào qiú    ***nǎgè   tóngxué***
        *the    boy         want       beg  which  classmate*
        解决  问题?
        jiějué  wèntí?
        *solve problem*
        ‘Which classmate does the boy want to beg to solve
        the problem?’
        b. *Declarative with indefinite object noun phrase*
        那个 男生       想要      求    一个 同学
        Nàgè nánshēng xiǎngyào qiú ***  yígè   tóngxué***
        *the    boy         want       beg  a       classmate*
        解决  问题.
        jiějué  wèntí.
        *solve problem*
        ‘The boy wants to beg a classmate to solve the problem.’
        c. *Declarative with definite object noun phrase*
        那个  男生       想要      求    那个   同学
        Nàgè  nánshēng xiǎngyào qiú ***  nàgè   tóngxué***
        *the     boy          want     beg  the      classmate*
        解决  问题.
        jiějué  wèntí.
        *solve problem*
        ‘The boy wants to beg the classmate to solve the problem.’

The results show that, when the *wh*-phrase is encountered, *in-situ* questions with a complex *wh*-phrase in Mandarin are slower at the *wh-*determiner position of the *wh*-phrase *nǎgè* “which” than their declarative counterparts containing an indefinite (i.e., *yígè “a”*). Furthermore, the slowdown carries on to the following noun region [i.e., *tóngxu*é “classmate” in (18)]. At this noun, the definite declarative is still slower than the indefinite declarative.

Based on these results, Pablos et al. (submitted) concluded that *in-situ* questions with a complex *wh*-phrase are processed significantly slower than declaratives with an indefinite noun phrase *at the whole phrase*; while they are only processed significantly slower than declaratives with a definite noun phrase *at the noun position*. These researchers connect processing differences at the *wh*-word *nǎgè* “which,” to the discourse-link (Pesetsky, 1987; Avrutin, 2000) related cost, a well-known fact in the processing literature (see De Vincenzi, 1996; Kaan et al., 2000; Donkers et al., 2013; and for opposite claims see Frazier and Clifton, 2002; Hofmeister and Sag, 2010, among others). This means that when no prior context is given, the discourse-link feature in *nǎgè* “which” leads to similar additional processing cost as that in the definite determiner *nàgè* “the” (assuming that definites are costlier than indefinites as discussed by Warren and Gibson, 2002). In contrast, no additional processing cost is found when processing indefinite *yígè* “a” because the indefinite does not require prior discourse information.

#### General discussion of experiments on mandarin Chinese *in-situ wh*-questions

The results from the processing of *in-situ* questions with a simplex and a complex *wh*-phrase in Mandarin Chinese show that, overall, both *wh*-phrase types (i.e., simplex and complex) are processed slower than *the indefinite noun phrases* within declaratives (i.e., *rén* “someone/person” and *yígè tóngxu*é “a classmate”), but these effects show different timing properties depending on whether the *wh*-phrase is complex or simplex.

Based on the hypotheses put forth in the section Test Case 2 for *wh*-question formation strategies across languages, the results obtained by Pablos et al. (submitted) for the processing of *in-situ* questions containing complex and simplex *wh*-phrases in Mandarin support the approach in which the question interpretation is only considered when the *wh*-phrase is encountered, and not before. Nevertheless, this prediction seems to only be met when differences between *in-situ wh*-questions and declaratives containing indefinite noun phrases are taken into consideration. Declaratives that contain definite noun phrases do not seem to pattern accordingly. Researchers have previously identified the reading time cost of proper names and definite noun phrases over indefinite noun phrases in the processing literature (see Warren and Gibson, 2002; Yen, 2007). Thus, this result is consistent with previous findings.

### General discussion of experiments on test case 2

In the four self-paced reading experiments on the processing of *in-situ* simplex and complex *wh*-questions in French and Mandarin Chinese, results show that both simplex and complex *wh*-questions are generally processed slower than declaratives with indefinite noun phrases. Overall, the results suggest that, as hypothesized by one of the processing strategies discussed in Test Case 2, speakers of French and Mandarin do not seem to consider the *in-situ wh*-question interpretation of the sentences until they encounter the *wh*-word/phrase. This seems to occur regardless of whether the language has different *wh*-question formation strategies or whether the only available strategy is the *in-situ wh*-question formation.

This suggests that the same processing mechanism is used in these two languages when no prosodic or contextual information is being considered. Furthermore, the results are compatible with the theoretical analyses of *in-situ wh*-questions involving covert dependencies between the *in-situ* item and the left-periphery.

As seen in the previous sections on the Mandarin and French experiments, we matched the experimental paradigms that we used for French and Mandarin as closely as possible bearing in mind the differences between the two languages. This strong parallelism provided us with the opportunity to see which effects were maintained across languages despite their differences and which effects could connect to the restrictions imposed by the research question that we pursued and the experimental technique we used. For example, the timing and length of the observed effects did not always coincide for both languages. This is very likely to be dependent on specific characteristics of the language and the data used, which point to several processes occurring at same time (e.g., dependency completion, referential assignment, etc.). The measurement of the effects by means of reading time differences can therefore not be conclusively associated to a single processing task, but might be connected to several other processes involved in the on-line comprehension of these constructions. Nevertheless, if we consider the overall result, the observable differences confirm the presence of on-line incremental interpretational processes in both languages. The results suggest that in both languages, the parser does not postulate the possibility of a question operator in CP before encountering the *in-situ wh* expression. Furthermore, the evidence coming from a close comparison of the two languages points to the existence of a common processing strategy adopted by their speakers.

## General discussion

In the previous sections, we have discussed two ways to conduct strongly theoretically informed experimental studies. In the first test case, we examined the processing of backward dependencies using two different linguistic phenomena (the referential interpretation of cataphoric pronouns and NPI licensing), with one method and one language. In the second test case, we examined the processing of one linguistic phenomenon (*in-situ wh*-questions) in different languages using a uniform method of testing and as closely as possible matched linguistic paradigms. The objective of these two tests cases was twofold: (1) to assess whether we can find common strategies in the processing of different backward dependencies and (2) to investigate whether there is a common strategy in how *wh*-*in-situ* questions are processed across languages.

Considering the evidence provided by the test cases discussed within this article, we can draw two major conclusions: (1) that the parser respects grammatical constraints, which means it is sensitive to differences in (hierarchical) structure, and (2) that there is a common parsing procedure for *in-situ wh*-question parsing phenomena in languages with different question formation strategies, where the analysis of the sentence as a *wh*-question does not seem to be assumed until overt evidence such as the *wh*-word/phrase is found in the input.

Based on what we have discussed so far, the question that remains is how our experimental results can feed theoretical linguistics or what insight can we gain from these results. In other words, how can our results contribute to the linking hypothesis discussed by Embick and Poeppel (2015). There are two possible reasons why this research can be relevant for theoretical linguistics. The first is more straitghforward, as it is connected to testing the same phenomenon in different languages with different question formation options. If the existing question formation strategies in these languages do not seem to make any difference for their parsing, then it means that at some level they share some basic properties. The main syntactic analyses of *in-situ wh*-questions assume a covert dependency (either through covert movement or a question operator binding with the *in-situ* element). The reported results are consistent with the establishing of a covert dependency (without choosing the particular type of ways to establish the covert dependency). The second is a more challenging one, since it comes from phenomena that are conceptually the same but different in their realization. The argument here would be that, if we find that the parser responds similarly to hierarchical relations, despite differences in the configuration of each tested structure, then it has to be the case that the parser can extract general grammatical properties out of specific input and that it can deduce the structural hierarchy behind the linearly presented input.

As discussed in the discussion of the challenges for theoretically informed experimental research in linguistics, there is usually some simplification of the theoretical question when searching for a suitable experimental paradigm. In our test cases, the starting theoretical question is much more complex than the evidence that we obtain, which supports there being hierarchical relations, for example. This means that, as researchers, we have to be aware of there being some theoretical questions that we are not going to be able to address yet. In particular, when we consider the relative maturity of the field of experimental linguistics, our current insight on experimental methods and procedure, there still exists a margin between the pursued theoretical question and the obtained results, i.e., the so-called *Granularity Mismatch Problem* in Poeppel and Embick's (2005) terms.

Finally, on the empirical side, our results are in line with current research that is connected to strongly theoretically based questions, such as the processing of Strong and Weak Crossover dependencies. For example, the research by Kush et al. (2017) also tries to examine how an incremental parser might interpret dependencies that can only be made licit once the right-hand of the sentence is known, which is comparable to the experiments on the processing of *wh*-*in-situ* questions. This is crucial when we compare this type of dependencies with the backward dependency cases, where the expectation for a licensor is turned into a forward search. This implies that backward and forward processes engage different parsing processes: in the case of backward dependencies there is a search for the licensor started at the licensee (the pronoun or NPI in our test case 1), whereas in the *in-situ* questions there is a retrieval or backward search for a licensor started at the licensee (the *wh*-word/phrase). There is an overall tendency in the field of psycholinguistics to compare the processing of dependencies with similar characteristics in terms of retrieval and attraction processes in order to shed further light into how closely the parser follows the constraints of grammar. Work from Parker and Phillips (2016, 2017), for example, has compared licensor-NPI, reflexive-antecedent and subject-verb agreement dependencies in an attempt to investigate how much these dependencies look alike in their parsing routines. Our first test case on the processing of backward dependencies connects with this research in that dependencies that seem apparently quite different in their realization can show a similar processing behavior.

To conclude, it seems to us that the only way to reach some maturity in the field of experimental linguistics research is to generate a big pool of evidence that builds upon showing some of the basic properties of language in performance across different languages, so that, with time, it will be possible to find evidence for more complex relations, enabling us to bring theory and experimental evidence closer.

## Author contributions

LP, JD, and LC conceived the project, were involved in all aspects of the design of the proposed methodology as well as on the interpretation of the results. LP was involved in the experiment creation and implementation, data analysis and contributed to drafting the manuscript. JD and LC critically revised the manuscript. All authors are responsible for final approval of the version to be published.

### Conflict of interest statement

The authors declare that the research was conducted in the absence of any commercial or financial relationships that could be construed as a potential conflict of interest. The reviewer, EC, and handling Editor declared their shared affiliation.

## Footnotes

^1^Negative polarity items are items such as *anything* in English, which must appear under certain licensors, such as negation, as we can see from the comparison between (ia) and (ib):

(i) a. John didn't buy anything.
     b. ^*^John bought anything.

^2^At the time, there existed some ERP studies on the processing of forward (antecedent-)pronoun configurations (see Osterhout and Mobley, 1995; Van Berkum et al., 2007; Xu et al., 2013), but little evidence existed about how backward pronoun dependencies were processed in ERP terms. Studies on forward pronoun dependencies have resulted in the generation of a P600 at the mismatched pronoun *she* in contrast to the matched pronoun *he* in configurations such as the one in (i) from Osterhout and Mobley (1995). (i) **The uncle** hoped that **he/she** had picked a good wine.

^3^The indexing of (5) indicates the intended reading and abstracts away from the possibility that the pronoun *haar* “her” has a referent that is not mentioned in the sentence. This is also a possibility, in particular when the sentence is embedded in a context in which the referent has already been mentioned. In the experiment, the examples were given to the participants without further context. Our results, and the results of Kazanina et al. (2007) show that the parser starts an active search for a referent within the sentence after the pronoun is encountered.

^4^The P600 is an Event-related component with positive polarity whose onset usually occurs around 500–600 ms and which peaks around 600 ms. Its topographical distribution is strongest over centro-parietal electrodes. It is a component that is generally elicited by syntactic violations, and its amplitude reflects the degree of difficulty of syntactic integration or reanalysis in parsing (see Osterhout and Holcomb, 1992; Hagoort et al., 1993; Kaan et al., 2000; Friederici et al., 2001, among others). The reader is referred to the discussion in Pablos et al. (2015) for the potential reasons for the lack of a P600 in the gender mismatch comparison in (5) and (6) at the antecedent.

^5^According to Hoekstra (1991) and Hoeksema (2000, p. 25), fronting a clause with a NPI in it yields grammatical results. Both argue that this is due to reconstruction at Logical Form, which places clauses back in their original positions. This further allows the complement-clause in (9a) to be within the scope of the matrix negator *niet* “not.” Following their account, in this study, we assume that the NPI under examination is within the scope of the matrix clause negation and thus licensed by it.

^6^As we discuss in section Test Case 2, Warren and Gibson (2002) showed that the nature of the referring expression that has to be intergrated while a syntactic dependency or prediction is maintained can increase the syntactic complexity of the sentence. For example, indexical first and second person pronouns generate less syntactic complexity than indefinite or definite noun phrases, since the latter refer to less accessible discourse entities.

^7^We consider that the content of these modifiers will not disrupt the on-line interpretation of the sentence, in particular in the search for a licensor once the NPI is parsed. In this sense, even if the modifiers are different in nature from parentheticals, we follow the assumptions made by work that used parentheticals to extend the linear distance of the elements within a long-distance dependency (see Dillon et al., 2014; Parker and Phillips, 2016, for examples).

^8^Notice that there is a problem inherent to the design and to the central question of our experiment and that is that we will never be able to match all the conditions closely, since they all differ in the number of words and modifiers. One potential solution would be to look at the ERP modulation of the whole sentence in a similar manner to Phillips et al. (2005), Fiebach et al. (2002), or King and Kutas (1995).

^9^Both (13a,b) are used in informal French only. In more formal registers, fronting is combined with subject-verb inversion or insertion of the question particle *est-ce que*. There are various pragmatic and grammatical differences between the fronting structure in (13a) and the in-situ one in (13b) as well as between the different possible fronting structures. For instance, the question word *pourquoi* is claimed to be bad in *in-situ* questions, while it is perfectly grammatical in most fronting questions, including the type illustrated in (13a) (Behnstedt, 1973). A full comparison between the different factors that may play a role in the choice between question strategies in French is beyond the scope of this article (but see, for instance, Boucher, 2010 for an overview).

^10^Proper Names are known to result in higher processing cost than other referential noun phrases (see Warren and Gibson, 2002 for further discussion). We chose Proper Names for our design because they were the definites that consisted of single words.

^11^In Mandarin Chinese proper names are at least bisyllabic. This is the reason why, bisyllablic proper names are used in the study even though their corresponding interrogative and indefinite counterparts are monosyllabic.

^12^*Nà* “that” in Chinese is a demonstrative which is often used without a distal demonstrative reading. In such cases, its reading is more like a definite article (see Huang, 1999).

^13^As discussed in footnote 11, in order to use completely natural stimuli for Mandarin, we could only use bisyllabic proper names.
